# Sexual networks, sexual practices, and sexual health among youths in WHO-South East Asia Region: a scoping review protocol

**DOI:** 10.1186/s13643-025-02905-0

**Published:** 2025-07-12

**Authors:** Amrita Rao, Rashmi Shinde, Sheikh Mohammed Shahabuddin

**Affiliations:** 1 Division of Clinical Sciences, ICMR-National Institute of Translational Virology & AIDS Research, 73 G Block MIDC, Pune, Maharashtra 411026 India; 2 Division of Disease Elimination Sciences, ICMR-National Institute of Translational Virology & AIDS Research, 73 G Block MIDC, Pune, Maharashtra 411026 India; 3 Library, ICMR-National Institute of Translational Virology & AIDS Research, 73 G Block MIDC, Pune, Maharashtra 411026 India

**Keywords:** Sexual network, Sexual practices, Sexual health, Youths, WHO-South East Asia Region

## Abstract

**Background:**

South-East Asia Region has one of the largest youth populations in the world. All countries are striving to achieve the sustainable development goal by 2030; hence, it is important to prioritize healthcare services for youths. Youths in the age bracket of 18 to 24 years often engage in high-risk behaviors such as unsafe injecting practices/substance abuse. These high-risk practices lead to increased transmission of sexually transmitted infections including HIV among them. It is imperative to understand the dynamics around sexual transmission of diseases among youth. This review will map the available evidence and identify the gaps in sexual health interventions related to the sexual networks, sexual practices, and sexual health among youths across the World Health Organization (WHO)-South East Asia Region (SEAR).

**Methods:**

The scoping review is guided by the Arksey and Malley framework. Peer-reviewed articles focusing on youths in the age groups of 18 to 24 years, from the 11 countries of SEAR, will be accessed from three databases, namely PubMed, Scopus, and Journals@Ovid. Additionally, grey literature from 2015 to date will also be accessed. Two reviewers will independently screen the articles based on pre-defined eligibility criteria in *Rayyan software*. Data extraction will be carried out based on pre-specified variables aligned with the objectives. We will synthesize the evidence from the relevant qualitative, quantitative, and mixed method studies. The reporting will follow Preferred Reporting Items for Systematic reviews and Meta-Analysis extension for Scoping Reviews (PRISMA-ScR).

**Discussion:**

This review will help to generate evidence focusing on the current sexual networks, sexual practices, and sexual health among youths in WHO SEAR with a focus on India, highlighting the gaps in sexual health interventions that need to be bridged. The insights from this review will assist in designing larger evidence-based intervention studies for improving the sexual networks, sexual practices, and sexual health among youths in this region. The findings from this review will be disseminated through peer-reviewed journals and conferences.

**Systematic review registration:**

Open Science Framework. The link is 10.17605/OSF.IO/2JSMC.

**Supplementary Information:**

The online version contains supplementary material available at 10.1186/s13643-025-02905-0.

## Background

Globally, young people who are in the age bracket of 10 to 24 years comprise 1.8 million; 16% of the total population [1]. Young people comprise of youths in the age bracket of 15 to 24 years and adolescents between the ages of 10 and 19 [2]. Young people are considered a priority group, and engaging them for community health strengthening will be a step closer to achieving the targets of sustainable development goals by 2030 [3]. Further, adolescence is associated not only with rapid changes in growth and development but also with an increased desire for independence, stronger peer influence, and a heightened tendency for risk-taking and experimentation. This could escalate their risk-taking ability for substance use, unprotected sexual activity, and mental health challenges during this critical developmental period.

In Bhutan and Bangladesh, some of the issues highlighted were that the program staff were reluctant to distribute condoms and talk about sexually transmitted infections and HIV testing to adolescent clients [4]. These inhibitions need to be resolved among the caregivers so that better care and services are provided to adolescents and extended to youths. The recent World Health Organization (WHO) guidance on accelerated action for adolescent health is based on the current health status, mortality, and morbidity. This will help planning and implementing strategies focusing on this age group [5]. While adolescent health care remains a focus, youths may be neglected. Further, in a conservative society, sex-related issues are a taboo for discussion; hence, youths do not actively seek counselling regarding sexual health. Social ostracism and disease-associated stigma have created an attitude of negativity and shame in the minds of this population. Vulnerability to high-risk behaviors, including substance abuse and high-risk sexual behavior such as lack of condom use and multiple sexual partners, often leads to increased chances of sexually transmitted infections (STI), including HIV [6, 7]. Youths may seek care at the adult health care facilities, and a few may not even approach these facilities for treating STIs. Obtaining sexual history, especially from youths, requires counselling skills in addition to clinical skills. Hence, gathering this history is a challenge at times. Partner seeking and history of sexual exposure among youths need to be sought in the privacy and comfort of the youth.

Recent estimates indicate that around 0.21 million new HIV infections were reported among adolescent girls and young women aged 15 to 24, while among adolescent boys and young men (15 to 24 years), the sub-Saharan region contributed to nearly 63% of these infections [8]. Developed countries like the USA are working to reduce disparities and improve HIV outcomes among youths aged 13 to 24, who account for nearly 21% of the 37,968 new HIV diagnoses [9]. Even in India, the recent NFHS-5 data revealed that among the unmarried women and men of age 15–29 years, 4.2% of women and 23.3% of men had more than one sexual partner, and 17.24% of men had paid sex [10].

A few studies across the world have revealed that youths have poor knowledge of sexual and reproductive health and many of them do not even access the services. It is a common practice to discuss these issues among their own peers [11, 12]. In addition, studies from various regions worldwide have highlighted a significant prevalence of sexual activity among unmarried adolescents and youths of both sexes. These studies indicate a decreasing age of sexual initiation, evolving sexual practices and preferences, and engagement in high-risk behaviors such as unprotected intercourse with multiple partners, raising serious public health concerns. Sexual behavior is shaped by a complex interplay of physiological factors, alongside rapidly shifting cultural and social influences that vary across generations [13, 14]. Recent technological advancements and modernization have expanded partner seeking beyond the physical locations into the virtual world [15], making partner tracing complex.

Hence, it is important to study the risk potential of infectious disease transmission among specific populations. Understanding sexual behaviors, tracing sexual partners, are an important focus to prevent the spread of infections. These interactions and understanding of this dynamics will help to develop interventions for specific population groups that will prevent the spread of infections from high-risk groups to low-risk populations. Studying these sexual and social networks has to be specific for each locality or environment, and it cannot be generalized across all populations. This is an essential component to help improve health, especially related to HIV and STI [16].

Based on this background, this review aims to conduct a scoping review on literature published on sexual networks, sexual practices, and sexual health among youths in the WHO South East Asia Region (SEAR).

The objectives of this review are as follows:

1. To comprehensively map the available evidence and understand the sexual networks, sexual practices, and sexual health among youths across the WHO-South East Asia Region (SEAR).
2. To identify research gaps to facilitate larger multi-centric projects related to the sexual networks, sexual practices, and sexual health among youths across the WHO South East Asia Region (SEAR).

## Methods

### Protocol design

The scoping review adopts an Arksey and O’Malley framework [17]. This framework involves the following steps: (1) formulating the research questions. (2) Developing the search strategy and identifying relevant articles. (3) Selecting eligible studies. (4) Extracting data. (5) Collating, organizing, and synthesizing the evidence. We have registered the protocol in Open Science Framework (OSF). The link is 10.17605/OSF.IO/2JSMC.

Preferred Reporting Items for Systematic Review and Meta-Analysis Protocols (PRISMA-P) checklist has been used while developing and reporting the protocol (Supplementary file 1) [18].

### Step 1: identifying the research question

The research question focuses on sexual networks, sexual practices, and sexual health among youths in WHO-South East Asia Region, as well as identifying and characterizing the gaps in the sexual health interventions. The following questions will guide the review.

1. What strategies/approaches are used to identify sexual networks, sexual practices and sexual health among youths in WHO-SEAR?
2. What are the gaps in existing sexual health interventions?

### Step 2: developing the search strategy and identifying the relevant articles

#### Eligibility criteria

The inclusion and exclusion criteria are defined in Table 1. Since we are looking into the current practices of sexual networks, sexual practices, and sexual health, we have restricted the search strategy to include studies conducted over the last decade (from 1 January 2015 till 31 December 2024). We will search articles from three databases: PubMed, Scopus, and Ovid (Journals@Ovid). Both published and grey literature will be accessed. We will access the grey literature reports through the stakeholders and the experts during our consultations, and also, if accessible, from the World Health Organization during the same time period. The review will include studies with quantitative, qualitative, and mixed-method study designs. Publications focusing on youths (18 to 24 years) and on the eleven countries of WHO-SEAR will be included. We shall include studies that involve a wide age range and multi-centric studies that have disaggregated data pertaining to youths (18 to 24 years) and from the WHO SEAR countries. We shall include studies that are published only in English or that are translated into English since the team is fluent in only the English language.

**Table 1:** Eligibility criteria

	Inclusion criteria	Exclusion criteria
Publication date	1 January 2015 till 31 December 2024	Prior to 1 January 2015
Language	Only in English	Other languages
Objectives	Focus on sexual networks, sexual practices, and sexual health	Antenatal care, maternal health, postnatal care, neonatal care, and immunization will not be considered
Population	Age between 18 and 24 years	Those not in this age group
Settings/context	Only those studies from the 11 WHO SEAR countries	Those not in the 11 countries under WHO SEAR

#### Conceptual framework and definition

In this review we will use the following three definitions:

Sexual networks “are the network structures that emerge when individuals have sexual contact with each other” [20].

Sexual practices “are those actions, which people define as sexual, and their relationship to the architecture of society. These practices vary widely between and within societies, and change significantly through time” [21].

Sexual health “a state of physical, emotional, mental and social well-being in relation to sexuality; it is not merely the absence of disease, dysfunction or infirmity. Sexual health needs a positive and respectful approach to sexuality and sexual relationships, as well as the possibility of having pleasurable and safe sexual experiences, free of coercion, discrimination and violence. For sexual health to be attained and maintained, the sexual rights of all persons must be respected, protected and fulfilled” [22].

The search terms will focus on the broader domains of “sexual networks,” “sexual practices”, “sexual health”,  “gaps in sexual health intervention”, “youths”, and the “11 countries belonging to WHO SEAR*.*” This strategy will be developed by the core team, which includes two subject experts and will be peer-reviewed by the librarian (information specialist). Each domain will be conceptualized using PCC (population, concept and context) framework, followed by identifying relevant search terms for each element of the framework.

*Type of population*—youths in the age bracket of 18 to 24 years.

*Types of concept*—we have identified three broad domains, namely sexual network, sexual practices, and sexual health. Each domain is fragmented into different concepts. Sexual network is fragmented into concepts like virtual network, sexual and social network, sexual partnerships, and sexual relationship. Sexual practices are broken into concepts like sex education, advocacy, communication, behavioral interventions, and behavioral risk. Sexual health focuses on the concepts like peer education and awareness around youth-friendly health services.

*Types of context*—*we* will search for studies that focus on the 11 member countries of the WHO South-East Asia Region, namely Bangladesh, Bhutan, Myanmar, India, Indonesia, Maldives, Nepal, Sri Lanka, Thailand, Timor-Leste, and the Democratic People’s Republic of Korea.

#### Search strategy

A librarian, with experience in data management and literature retrieval, will help develop the search strategy and also access relevant databases such as PubMed, Scopus, and Journals @Ovid (through Ovid platform) for data retrieval. While PubMed is a platform offering access to biomedical literature, especially citations from Medline indexed journals and papers in PubMed Central, Scopus provides access to indexing, abstracting, and citation data of scientific literature across various disciplines. Besides, Ovid platform allows access to Journal@Ovid database, which indexes journals from 50 publishers and societies. For our study, we have considered young adults in the age bracket of 18 to 24 years. However, in PubMed, we have included articles that define “young adults” as individuals aged 19 to 24 years. For the other two databases, we have used the term youths and young adults to retrieve articles. The search is structured using the Joanna Briggs Institute(JBI) recommended PCC (population/concept/context) framework. Every element of the framework is clearly defined using keywords and index terms (such as MESH terms in PubMed) so that they can be used along with Boolean operators to search different databases effectively. The search strategy used to search PubMed is given (Supplementary file 2). The Preferred Reporting Items for Systematic Reviews and Meta-Analysis extension for Scoping Reviews (PRISMA-ScR). PRISMA-ScR will be followed while reporting [19].

### Step 3: selecting eligible articles

The search results from all the databases will be imported into *Rayyan (*(https://rayyan.ai/). This will be followed by removing duplicates, followed by title and abstract screening in *Rayyan*. This will be done by two reviewers independently to select studies that are relevant and fit the definition, inclusion criteria fitting into the PCC framework (population/concept/context). The articles that have been selected during the title and abstract screening will undergo full text screening. All relevant articles, qualitative, quantitative, and mixed-method studies will be included. Mixed-methods studies will be considered if the data from the quantitative or qualitative components can be extracted separately. The reasons for excluding the articles will be noted. Data extraction will be done by two independent reviewers. Any conflicts of opinion between the reviewers will be resolved mutually or, if needed, by the third person (co-author). The final search results will be reported in the PRISMA flow diagram (Supplementary file 3). The study is limited to the articles, which are published in the English language.

### Stage 4: extracting data

Two independent reviewers will be involved in the data extraction. A data extraction form adopted from Joanna Briggs Institute’s template will be used for data extraction [23]. The key information collected from relevant studies will include date of publications, country/state, study design, objectives, methodology, and results. The data extraction will cover the details on the participants, including the socio-demographic profile and inclusion and exclusion criteria. The central concept of the study, including the operational definition if any, will be extracted. The socio-cultural, institutional, and economic context information will also be collected. We will also note the relevant findings and outcomes in terms of the PCC framework. The quantitative outcomes will represent the knowledge, awareness, and other factors related to sexual practices and sexual health, such as the risk of sexually transmitted infections and partner tracing. The data extracted from the qualitative component will include verbatim extract, interpretation of the results, and illustrations.

The data extraction form is prepared and will be pilot tested (Supplementary file 4). Data extraction agreement will be performed at regular intervals of 10%, 50%, and after completion of the selected studies. This would be done in consultation with all the team members. Necessary adjustments and changes will be made in consultation with the team members. Since this is a scoping review, quality assessment of articles is not mandatory.

### Stage 5: collating, organizing, and synthesizing the evidence

In this stage, the scoping review will synthesize evidence on sexual networks, sexual practices, and sexual health among youth in the WHO South East Asia Region. This will help identify the current sexual health interventions and the existing gaps to help countries draw upon the interventions that have worked and that have not worked for youths. This will play an important role in identifying factors that prevent the transmission of STIs, including HIV. It will also support the development of mechanisms to identify and trace sexual networks, increase the uptake of sexual health services, and create awareness among the youths.

Data extraction will be done using MS Excel. The outcomes from these studies will be tabulated and summarized narratively. For quantitative studies, the summary of findings and descriptive analysis will be reported based on the type of data extracted. Descriptive statistics will be used to present the findings. We shall try to pool the data and conduct meta-analysis on studies with common outcomes. Where statistical pooling is impossible, the findings will be presented in narrative form, including tables and figures where applicable, to aid in data presentation. For the qualitative studies, we will use charts to identify different concepts and maps of descriptive themes to populate analytical domains. This framework will be modified based on the emerging themes during analysis and in consultation with the researchers in the team. Coding of data will be conducted based on the themes identified. Each study will be indexed based on the themes identified. If newer themes emerge irrespective of their reconciliation with findings from other studies, they will be included in the interpretative framework.

The synthesized quantitative and qualitative findings will be integrated using a configurative analysis. These findings will be juxtaposed against each other to organize or link the evidence generated.

## Discussion

This review is an effort to summarize the evidence around sexual networks, sexual practices, and sexual health among youths in the WHO-SEAR. Generating evidence around sexual networks, sexual practices, and sexual health among youths will help gain insights into the current sexual networks, sexual health, and sexual practices among youths. This comprehensive understanding will help to develop appropriate interventions.

This scoping review may miss articles that focus on adolescents, as we have looked into youths in the age bracket of 18 to 24 years in the WHO-SEAR member countries. We shall not undertake the quality assessment as it is not mandatory for a scoping review. This may limit the strength of the evidence generated. Additionally, relevant articles in other languages will be missed as we have included articles published in English language only. Furthermore, we will not take into consideration published protocols, case reports, and systematic reviews even though they focus on sexual networks, sexual health, and sexual practices in WHO SEAR countries.

## Supplementary Information

Supplementary Material 1. PRISMA-P (Preferred Reporting Items for Systematic review and Meta-Analysis Protocols) 2015 checklist: recommended items to address in a systematic review protocol*.

Supplementary Material 2. Draft search strategy.

Supplementary Material 3. PRISMA flowchart.

Supplementary Material 4. Data extraction form.

## Data Availability

Not applicable.

## Abbreviations

HIV: Human immunodeficiency virus
OSF: Open Science Framework
PCC: Population /Concept/Context
PRSIMA-ScR: Preferred Reporting Items for Systematic Reviews and Meta-Analyses Extension for Scoping Reviews
SEAR: South East Asia Region
STI: Sexually transmitted infections
WHO: World Health Organization
