# Supplementary material for: Sexual networks, sexual practices, and sexual health among youths in WHO-South East Asia Region: a scoping review protocol
Source: Syst Rev. 2025 Jul 12;14:147. doi: 10.1186/s13643-025-02905-0 (PMC12255040; doi:10.1186/s13643-025-02905-0)
Supplement: Supplementary file 2 — Supplementary Material 2. Draft search strategy. [file 13643_2025_2905_MOESM2_ESM.docx]

Supplementary file 2: Draft search strategy

PubMed search as on 31^st^ Dec 2024

| **Search No.** | **Search terms** | **Result** |
| --- | --- | --- |
| 1 | "Sexual Partners"[Mesh] OR “Sexual networks” OR “Virtual network” OR “Sexual & social network” OR “Sexual partnership” OR “Sexual relationship” OR “Sexual partners” OR “Dating apps” OR “Networking apps” OR “Social websites” OR “Adult websites” OR Sexting | 34268 |
| 2 | "Sex Education"[Mesh] OR “Sex education” OR "Sexual Behavior"[Mesh] OR "Sex Work"[Mesh] OR “Sexual practices” OR “Sexual behavior” OR “High-risk practices” OR “Drug-sex interface” OR Chemsex OR “Sex seeking” OR “Sex solicitation” OR “Dating” OR “Commercial sex” OR “Casual sex” OR “Pre-marital sex” | 187622 |
| 3 | "Sexual Health"[Mesh] OR "Reproductive Health Services"[Mesh] OR "Health Services"[Mesh] OR “Reproductive Health Services” OR “Health Services” OR “Sexual Health Services” OR “Health services uptake” OR “Sexual and reproductive health service” OR “Sexual health” OR “Youth Friendly Health services” | 2777550 |
| 4 | #1 OR #2 OR #3 | 2938468 |
| 5 | "Contact Tracing"[Mesh] OR “Contact tracing” OR “Partner notification” OR “Sexual exposure” | 10660 |
| 6 | "Safe Sex"[Mesh] OR "Unsafe Sex"[Mesh] OR “safe sex” OR “Unsafe sex” OR Availability OR Accessibility | 2411993 |
| 7 | "Social Stigma"[Mesh] OR "Sexism"[Mesh] OR "Social Discrimination"[Mesh] OR "Perception"[Mesh] OR Reach OR “Social Stigma” OR “Social discrimination” OR Barriers OR Facilitators OR Uptake OR “non-uptake” OR Apprehensions OR Experiences OR Perceptions OR Hesitancy OR Hindrance OR Deterrent OR Constraints OR Obstacles | 4135938 |
| 8 | #5 OR #6 OR #7 | 6057892 |
| 9 | #4 AND #8 | 759652 |
| 10 | #4 AND #8 and Filters: Young Adult: 19-24 years | 74733 |
| 11 | Bangladesh OR Bhutan OR Myanmar OR Burma OR India OR Indian OR Indonesia OR Maldives OR Nepal OR “Sri Lanka” OR Thailand OR “Timor-Leste” OR “East Timor” OR “Democratic Republic of Timor-Leste” OR “North Korea” OR “Democratic People’s Republic of Korea” | 1277409 |
| 12 | #10 AND #11 | 3908 |
| 13 | Search: **#10 AND #11** Filters: **from 2015/1/1 - 3100/12/12** | 2610 |
