# Supplementary material for: Sexual networks, sexual practices, and sexual health among youths in WHO-South East Asia Region: a scoping review protocol
Source: Syst Rev. 2025 Jul 12;14:147. doi: 10.1186/s13643-025-02905-0 (PMC12255040; doi:10.1186/s13643-025-02905-0)
Supplement: Supplementary file 3 — Supplementary Material 3. PRISMA flowchart. [file 13643_2025_2905_MOESM3_ESM.docx]

Supplementary file 3 : PRISMA flowchart

**Identification**

**Eligibility**

**Screening**

**Included**

( ) duplicate records removed

( ) Records after removal of duplicates

( ) Records identified through searching PubMed ( ), Scopus ( ) and Journals@Ovid ( )

( ) Records excluded

( ) records finally obtained from bibliographic databases

**( )** articles excluded

( ) Not the correct population

( ) wrong study site

( ) Full-text articles assessed for eligibility

( ) Records screened in Rayyan

**( )** records included in synthesis
