# Supplementary material for: Sexual networks, sexual practices, and sexual health among youths in WHO-South East Asia Region: a scoping review protocol
Source: Syst Rev. 2025 Jul 12;14:147. doi: 10.1186/s13643-025-02905-0 (PMC12255040; doi:10.1186/s13643-025-02905-0)
Supplement: Supplementary file 4 — Supplementary Material 4. Data extraction form. [file 13643_2025_2905_MOESM4_ESM.docx]

Supplementary file 4: Data extraction Form

1. S. No.
2. Authors
3. Title of the article
4. Name of the Journal
5. Month & year of Publication
6. Volume
7. Issue
8. Pages
9. Type of article : Qualitative/ quantitative/mixed method
10. Aim/objectives of the study
11. Study duration
12. Study design: cross-sectional/ cohort/ retrospective/ prospective/ randomized control trial/ non-randomized control trial
13. Study site: country/ state/ rural./ urban
14. Study participants –number of participants/ enrolment process
15. Context of the study- with the objectives of the review
16. Implementation of newer strategies/ Newer interventions (if any)
17. Current gaps
18. Findings: focus on sexual networks/ sexual practices/ sexual health/ trace participants/ access to partners/ sexual health facilities
